# Hemiarch repair versus total-arch repair with frozen elephant trunk for acute type A aortic dissection: A propensity score–matched study

**DOI:** 10.1016/j.xjon.2026.101945

**Published:** 2026-06-30

**Authors:** Julin Zhang, Jili Li, Yaoye Chen, Xingrui Tao, Qianlei Lang, Zhefeng Kang, Jiuhong Li, Hao Niu, Xiangfeng Gong, Ruofan Zhou, Jia Hu, Wei Meng, Zhong Wu, Michael W.A. Chu, Zhenghua Xiao, Chaoyi Qin

**Affiliations:** a Department of Cardiovascular Surgery and Cardiovascular Surgery Research Laboratory, West China Hospital, Sichuan University, Chengdu, Sichuan, China; b Department of Thoracic Surgery, West China Hospital, Sichuan University, Chengdu, Sichuan, China; c West China School of Medicine, West China Hospital, Sichuan University, Chengdu, Sichuan, China; d Division of Cardiac Surgery, Department of Surgery, Western University, London Health Sciences Centre, London, Ontario, Canada

**Keywords:** acute type A aortic dissection, hemiarch replacement, TAR with FET, aortic remodeling, aortic reintervention

## Abstract

**Objective:**

The optimal extent of arch repair remains controversial in acute type A aortic dissection. Although hemiarch replacement (HAR) may offer more favorable perioperative outcomes, total arch replacement with frozen elephant trunk (TAR with FET) may offer superior long-term outcomes. This study aims to compare these strategies in a contemporary single-center cohort.

**Methods:**

Patients undergoing arch repair for acute type A aortic dissection between January 2020 and January 2025 were included. Propensity-score matching was used for further analyses. Primary outcomes included operative mortality and aortic reinterventions. Secondary outcomes included re-exploration for bleeding, renal failure, respiratory failure, neurologic complications, and aortic positive remodeling, defined as true lumen expansion greater than 50%.

**Results:**

Among 786 patients (171 hemiarch, 615 total arch), 272 patients were included in the matched cohort, comprising 136 matched pairs of HAR and TAR with FET, with a median follow-up of 3 years. Perioperative outcomes were comparable: operative mortality (5.1% HAR vs 7.4% TAR with FET; odds ratio [OR], 1.50; *P* = .442), renal failure (12.5% vs 8.8%; OR, 0.71; *P* = .353), respiratory failure (22.8% vs 25.7%; OR, 1.17; *P* = .579), and neurologic complications (13.2% vs 16.2%; OR, 1.29; *P* = .481). Meanwhile, 3-year survival (*P* = .420) and aortic reintervention rates (*P* = .135) were also comparable. Computed tomography angiography demonstrated superior aortic remodeling after TAR with FET replacement, with a greater true lumen ratio and a greater rate of true lumen expansion (28.7% vs 42.9%; OR, 0.54; *P* = .019). In a subgroup analysis of patients with acute malperfusion, perioperative mortality and major complications remained high overall. However, no significant differences were observed between HAR and TAR with FET in this subgroup.

**Conclusions:**

HAR and TAR with FET were associated with similar perioperative outcomes and 3-year survival rate. Although aortic remodeling is superior in TAR with FET group, 3-year aortic reintervention rates were comparable. Our findings suggest that TAR may be considered more proactively in high-volume aortic centers; however, longer-term clinical benefits remain undefined. Further study is warranted.

## Graphical Abstract

**Figure:**
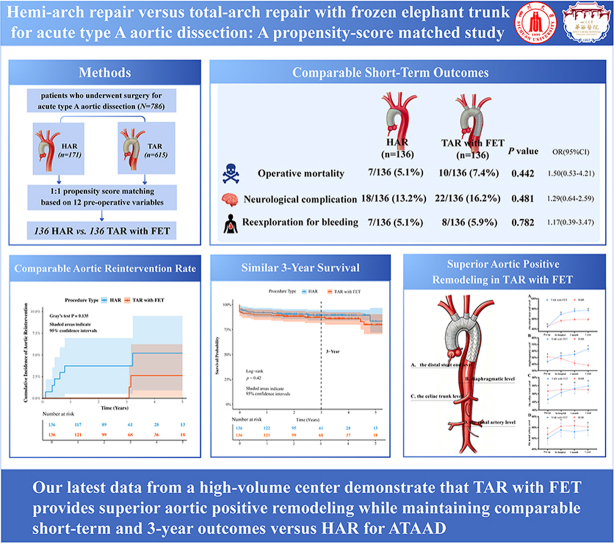

**Figure:**
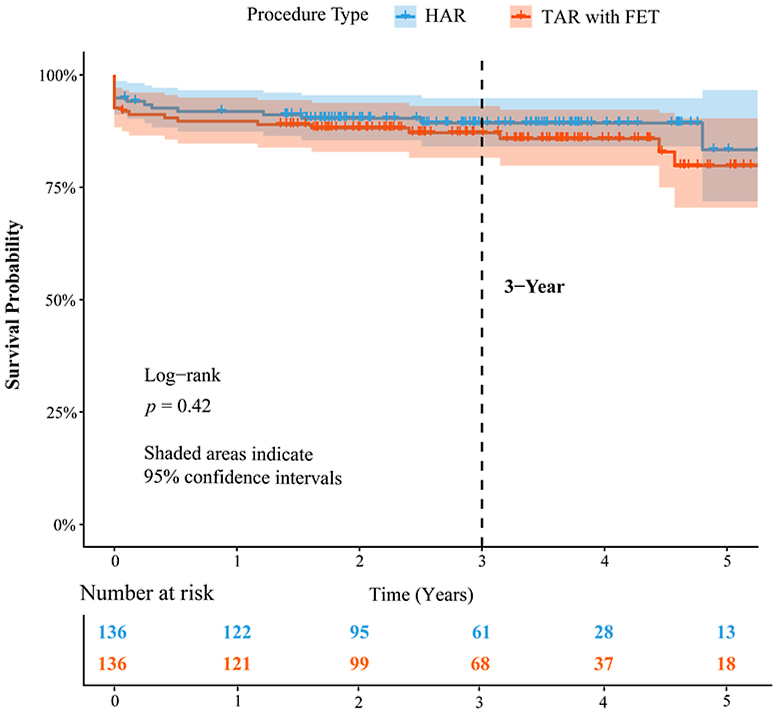
Comparable 3-year survival after HAR versus TAR with FET for ATAAD.

**Central Message**

TAR with FET demonstrated better aortic remodeling whereas perioperative outcomes, 3-year survival, and reintervention were similar to HAR in acute type A aortic dissection.

**Perspective**

In acute type A aortic dissection, TAR with FET achieved comparable perioperative outcomes and 3-year survival. Although TAR with FET showed superior aortic remodeling, the reintervention rate was similar between HAR and TAR with FET. These findings support a more proactive use of TAR with FET in experienced aortic centers, and long-term follow-up is needed to refine patient selection.

Acute type A aortic dissection (ATAAD) is a life-threatening cardiovascular emergency characterized by rapid progression, with mortality increasing at an estimated rate of 1% to 2% per hour after onset.^1^, ^2^, ^3^ Currently, 2 principal surgical strategies for arch repair are commonly adopted: hemiarch replacement (HAR) and total arch replacement (TAR) with frozen elephant trunk (FET).^4^

HAR has traditionally been regarded as the standard approach for many patients with ATAAD because it is technically less complex, expedient in the emergency setting, and effective in resecting the proximal entry tear.^5^, ^6^, ^7^, ^8^, ^9^ However, with advances in perioperative management and improved early survival, the long-term outcomes of the residual aortic arch after HAR are now receiving more attention.^10^^,^^11^

To improve the long-term outcomes of patients with ATAAD, TAR with FET has been increasingly used as a more aggressive surgical strategy. In Asia, more extensive aortic arch repair has been more commonly adopted for ATAAD in many centers.^7^^,^^12^^,^^13^ TAR with FET may provide more extensive treatment of arch pathology and potentially improve aortic remodeling. In addition, TAR with FET may help provide more aggressive treatment in patients with malperfusion.^14^ However, its long-term impact on aortic events and reoperation remains uncertain.^15^, ^16^, ^17^ At the same time, TAR with FET is technically more demanding and may be associated with a greater risk of perioperative neurologic complications, including stroke and spinal cord ischemia.^18^, ^19^, ^20^

Current observational studies remain a lack of high-quality evidence from large-scale, homogeneous patient cohorts to systematically compare the early safety and long-term efficacy of these 2 surgical strategies.^21^^,^^22^ To address this clinical controversy, this study retrospectively analyzed consecutive ATAAD cases from a large cardiovascular center, aiming to provide contemporary evidence.

## Methods

### Ethics Approval

The local institutional review board (study number: 2025-1706, approved on August 26, 2025) officially approved this study. The research was conducted in accordance with the principles of the Declaration of Helsinki. Because of the retrospective nature of the study, the requirement for informed consent was waived.

### Study Cohort

The present study was based on the West China hospital open Aortic Repair for Dissection (WARD) study. The WARD cohort was established in 2020, and all consecutive patients who underwent surgical repair for aortic dissection were enrolled. For the present study, we screened all patients with ATAAD who underwent arch repair between January 2020 and January 2025 at our center. The present comparative analysis and propensity-score matching were restricted to patients undergoing HAR and those undergoing TAR with FET. Patients treated with other arch strategies, such as zone 2 arch replacement and hybrid strategies, were not included in the final analytic cohort. Patients with isolated intramural hematoma or penetrating aortic ulcer were excluded. No age restrictions were applied. All procedures were performed at our institution by 5 established cardiovascular surgery teams. Perioperative management, including surgical, anesthetic, and perfusion support, followed standardized institutional practice. Postoperative care was provided in a dedicated cardiac intensive care unit by an experienced intensive care team specializing in the management of patients after cardiovascular surgery, including those with acute aortic dissection.

### Our Surgical Approach

We followed the fundamental principles of resecting the primary entry tear and restoring true lumen flow. Although there is no consensus about arch strategy for DeBakey I ATAAD, many surgeons at experienced aortic centers in Asia prefer TAR with FET. In our center, the final decision was determined by a comprehensive consideration of the patient's anatomical characteristics and general clinical status. Specifically, TAR with FET was more often selected in patients with an entry tear involving the aortic arch, those with DeBakey type I dissection, and those who were relatively younger or in better general condition, whereas HAR was more often favored in older patients or in those with more comorbidities.

Generally, 3 arterial lines (both upper limbs and one lower limb) and 2 temperature probes (nasal and bladder) are prepared. The surgery is performed via a median sternotomy approach. After systemic heparinization, cardiopulmonary bypass is established. The right axillary or femoral artery is the preferred site for arterial cannulation. Central cooling starts right after on pump, and the target temperature is 24 °C to 28 °C. After aortic crossclamp, we start from aortic root preparation and cerebral perfusion cannulation until the target temperature is approached.

Once moderate hypothermia is ready, we perform the distal part first and leave the root procedure to be completed during systemic rewarming. For HAR, we preferred retrograde cerebral perfusion through superior vena cava for cerebral protection. The dissected ascending aorta is resected until the origin of innominate artery, followed by the reinforcement with sandwich technique using felt strips or Dacron graft. A straight Dacron graft is used to connect to the aortic arch with an end-to-end anastomosis. The systemic perfusion is resumed after aortic crossclamp on the graft. The root procedure is performed during the rewarming and is anastomosed with previous graft before aortic declamping.

For TAR with FET, we prefer antegrade cerebral perfusion through direct innominate and/or left common carotid artery (LCCA) cannulation. The dissected ascending and arch are resected until zone 1 or zone 2 and the FET is deployed directly into the true lumen of the descending aorta. A 4-branched Dacron graft is anastomosed to the FET using 3-0 polypropylene running suture. Aortic crossclamp is applied on the Dacron graft and systemic perfusion is resumed through one Dacron branch. The LCCA is anastomosed to the Dacron branch first to restore both cerebral perfusions, followed by reconstruction of the left subclavian artery. The root procedure is performed during rewarming and is anastomosed to the previously 4-branched Dacron graft before aortic delcamping. The innominate artery is reconstructed while the heart resumes beating. Strategies (either preserved or replacement) for aortic root are dependent on the diameters and the involvement of dissection of the root, whereas the use of intraoperative transesophageal echocardiography illustrates the functioning of aortic valve.

### Cerebral Perfusion Strategies

The neuroprotection strategy is determined on the basis of moderate hypothermia (24 °C-28 °C). Unilateral or bilateral antegrade selective cerebral perfusion is primarily used through direct innominate artery and/or LCCA cannulation. The perfusion flow rate is maintained at 6 to 12 mL·kg^−1^ min^−1^, with the regulatory target of keeping regional cerebral oxygen saturation within ±10% of the baseline value. Retrograde cerebral perfusion was performed through the same superior vena cava cannula. The perfusion flow rate is maintained at 5 to 10 mL·kg^−1^ min^−1^, with the regulatory target of keeping regional cerebral oxygen saturation within ±10% of the baseline value.

### Outcomes of Interest and Clinical Follow-Up

The primary outcomes of interest were operative mortality and aortic reintervention. Secondary outcomes included re-exploration for bleeding, renal failure, respiratory failure, the incidence of neurologic complications, and true lumen expansion. Aortic reintervention included reoperations on the aortic root, aortic arch, or downstream aorta, such as the descending thoracic aorta or thoracoabdominal aorta. Neurologic complications were defined as ischemic stroke, hemorrhagic stroke, paraplegia, and other forms of neurologic dysfunction confirmed by imaging.

Aortic remodeling measurements were determined on the basis of computed tomography angiography (CTA) findings at 4 anatomical levels: the distal stent end, diaphragmatic, celiac trunk, and renal artery levels. True lumen expansion was defined as a true lumen diameter to the sum of true and false lumen diameters exceeding 50% at the diaphragmatic level on in-hospital postoperative CTA. True lumen diameters and the true lumen/(true lumen + false lumen) ratio were measured at each level during follow-up.

The median duration of clinical follow-up was 1068 days, and clinical follow-up was censored on October 10, 2025. Follow-up data on survival and aortic reintervention rates were primarily obtained through telephone interviews and postoperative outpatient clinic reviews.

### Statistical Analysis

Continuous variables were assessed for normality using the Shapiro-Wilk test. Normally distributed continuous variables are described as mean ± SD, and their between-group comparisons were performed using the paired *t* test; non-normally distributed continuous variables are described as median (interquartile range) and were compared with the paired Wilcoxon signed-rank test. Categorical variables are reported as number (percentage) and were compared using the McNemar test. Propensity-score matching was conducted in a 1:1 ratio using the nearest-neighbor method to balance the groups across 12 preoperative clinical variables. The 12 preoperative variables included in the propensity score model are detailed in Table E1. After matching, all 12 variables achieved standardized mean differences less than 0.2, suggesting that the baseline characteristics were well balanced between the 2 groups. The association between treatment and outcomes was measured using logistic regression, generating odds ratios (ORs) with 95% CIs. Survival analysis was performed using the Kaplan-Meier method with the log-rank test. All *P*-values were 2-sided, with an α-level of .05. Statistical analysis was performed using R, version 4.4.3.

## Results

### Baseline Characteristics

Before matching, the total cohort included 786 patients: 171 underwent HAR and 615 underwent TAR with/without FET. Baseline characteristics and perioperative outcomes in the unmatched cohort are summarized in Table E2. After 1:1 propensity-score matching, the final study cohort comprised 272 patients (136 pairs). All preoperative variables for the matched cohorts are summarized in Table 1.

**Table 1:** All assessed baseline variables after matching

Variable	Total (n = 272)	HAR (n = 136)	TAR with FET (n = 136)	*P* value
Sex, male, n (%)	188 (69.1)	86 (63.2)	102 (75)	.057
Age, y, mean (SD)	54.58 (11.06)	54.96 (11.85)	54.19 (10.23)	.415
BMI, mean (SD)	24.74 (3.32)	24.71 (3.25)	24.77 (3.39)	.876
Smoking, n (%)	103 (37.9)	44 (32.4)	59 (43.4)	.092
Hypertension, n (%)	202 (74.3)	100 (73.5)	102 (75)	.890
Renal insufficiency, n (%)	17 (6.2)	8 (5.9)	9 (6.6)	1.000
Diabetes, n (%)	11 (4)	7 (5.1)	4 (2.9)	.547
Marfan syndrome, n (%)	6 (2.2)	2 (1.5)	4 (2.9)	.683
Coronary artery disease, n (%)	9 (3.3)	4 (2.9)	5 (3.7)	1.000
Stroke, n (%)	5 (1.8)	0 (0)	5 (3.7)	.074
Valvular heart disease, n (%)	41 (15.1)	22 (16.2)	19 (14)	.719
Thyroid disease, n (%)	6 (2.2)	3 (2.2)	3 (2.2)	1.000
Arrhythmia, n (%)	7 (2.6)	2 (1.5)	5 (3.7)	.371
COPD, n (%)	13 (4.8)	8 (5.9)	5 (3.7)	.579
D-dimer, mg/L FEU, median [Q1, Q3]	5.74 [2.36, 13.62]	5.68 [2.04, 12.53]	5.91 [2.71, 14.23]	.675
Uric acid, mean (SD)	363.69 (137.91)	360.82 (145.02)	366.55 (130.89)	.754
Creatinine, μmol/L, median [Q1, Q3]	87.5 [69, 114.25]	84.5 [66.75, 111.25]	88.5 [72, 115.25]	.289
BNP, ng/L, median [Q1, Q3]	468 [183.25, 1096]	448.5 [171.25, 1163.5]	477 [192.75, 1061.75]	.953
CRP, mean (SD)	67.15 (65.03)	66.1 (65.93)	68.19 (64.35)	.799
Left ventricle, mm, median [Q1, Q3]	49 [46, 53]	49 [45, 53]	50 [46.75, 53]	.140
Previous cardiac surgery, n (%)	42 (15.4)	23 (16.9)	19 (14)	.584
Preoperative hypotension, n (%)	26 (9.6)	14 (10.3)	12 (8.8)	.831
Preoperative altered mental status, n (%)	30 (11)	16 (11.8)	14 (10.3)	.850
Preoperative limb ischemia, n (%)	32 (11.8)	18 (13.2)	14 (10.3)	.571
Retrograde aortic dissection, n (%)	7 (2.6)	3 (2.2)	4 (2.9)	1.000
Pericardial tamponade, n (%)	16 (5.9)	8 (5.9)	8 (5.9)	1.000
Entry tear, n (%)				
Ascending aorta	226 (83.1)	113 (83.1)	113 (83.1)	1.000
Aortic arch	32 (11.8)	17 (12.5)	15 (11)	.823
Descending aorta	8 (2.9)	4 (2.9)	4 (2.9)	1.000
No identifiable entry tear	6 (2.2)	2 (1.5)	4 (2.9)	.617

*HAR*, Hemiarch replacement; *TAR*, total arch replacement; *FET*, frozen elephant trunk; *SMD*, standardized mean difference; *BMI*, body mass index; *COPD*, chronic obstructive pulmonary disease; *FEU*, fibrinogen equivalent units; *Q1*, first quartile; *Q3*, third quartile; *BNP*, B-type natriuretic peptide; *CRP*, C-reactive protein.

### Operative Profiles

Significant differences in operative strategies were observed between the 2 groups (Table 2). Specifically, femoral artery cannulation (90.4% vs 75.7%, *P* = .003) and retrograde cerebral perfusion (50% vs 4.4%, *P* < .001) were more common in the HAR group. As expected, the TAR with FET was associated with significantly longer operative times, including cardiopulmonary bypass time (median: 210.5 minutes vs 184 minutes, *P* < .001), aortic crossclamp time (median, 160.5 minutes vs 120.5 minutes, *P* < .001), and circulatory arrest time (mean, 29.12 ± 9.53 minutes vs 19.93 ± 11.87 minutes, *P* < .001). Platelet transfusion was also greater in the TAR with FET group (*P* < .001). No significant differences were observed in other concomitant procedures or in transfusion requirements. Details of FET graft characteristics in the TAR with FET group was listed in the Table E3.

**Table 2:** Intraoperative details

Variable	Total (n = 272)	HAR (n = 136)	TAR with FET (n = 136)	*P* value
Operation duration, h, median [Q1, Q3]	7 [5.88, 7.9]	6.45 [5.4, 7.4]	7.25 [6.3, 8.06]	<.001
Cardiopulmonary bypass time, min, median [Q1, Q3]	197 [165, 240]	184 [150, 221.25]	210.5 [179.75, 253]	<.001
Crossclamp time, min, median [Q1, Q3]	144 [109.75, 179.25]	120.5 [100.75, 167.25]	160.5 [128.5, 193.25]	<.001
Arterial cannulation, n (%)				
Axillary artery	30 (11)	9 (6.6)	21 (15.4)	.038
Femoral artery	226 (83.1)	123 (90.4)	103 (75.7)	.003
Combined axillary and femoral artery	7 (2.6)	0 (0)	7 (5.1)	.023
Other	9 (3.3)	4 (2.9)	5 (3.7)	1
Cerebral perfusion, n (%)				
Unilateral antegrade	141 (51.8)	32 (23.5)	109 (80.1)	<.001
Bilateral antegrade	20 (7.4)	1 (0.7)	19 (14)	<.001
Retrograde	74 (27.2)	68 (50)	6 (4.4)	<.001
Circulatory arrest time, min, mean (SD)	24.53 (11.69)	19.93 (11.87)	29.12 (9.53)	<.001
Core temperature, °C, median [Q1, Q3]	24 [23.2, 24.6]	23.8 [23, 25.08]	24 [23.48, 24.4]	.241
Preserved aortic root, n (%)	126 (46.3)	61 (44.9)	65 (47.8)	.708
Aortic root replacement, n (%)	110 (40.4)	56 (41.2)	54 (39.7)	.899
Concomitant CABG, n (%)	30 (11)	12 (8.8)	18 (13.2)	.286
Packed red blood cells transfused, units, median [Q1, Q3]	0 [0, 2]	0 [0, 1.5]	0 [0, 2]	.2669
Fresh-frozen plasma transfused, mL, median [Q1, Q3]	0 [0, 300]	0 [0, 0]	0 [0, 362.5]	.103
Platelets transfused, units, median [Q1, Q3]	2 [1, 2]	1 [1, 2]	2 [1, 2]	<.001
Maximum intraoperative blood glucose, mmol/L, mean (SD)	11.09 (3.32)	11.03 (3.55)	11.15 (3.08)	.7717
Maximum intraoperative lactate level, mmol/L, mean (SD)	6.64 (3.84)	6.42 (3.78)	6.87 (3.91)	.3299

*HAR*, Hemiarch replacement; *TAR*, total arch replacement; *FET*, frozen elephant trunk; *Q1*, first quartile; *Q3*, third quartile; *CABG*, coronary artery bypass grafting.

### Outcomes

Postoperative variables for the matched cohorts are shown in Table 3. There were no statistically significant differences between the 2 groups in operative mortality (HAR group 5.1% vs TAR with FET group 7.4%; OR, 1.50; 95% CI, 0.53-4.21; *P* = .442) or aortic reintervention rates (4.4% vs 1.5%; OR, 0.33; 95% CI, 0.07-1.65; *P* = .179).

**Table 3:** Postoperative outcomes

Variable, n (%)	Total (n = 272)	HAR (n = 136)	TAR with FET (n = 136)	*P* value	Odds ratio (95% CI)
Operative mortality	17/272 (6.2)	7/136 (5.1)	10/136 (7.4)	.442	1.50 (0.53-4.21)
Re-exploration for bleeding	15/272 (5.5)	7/136 (5.1)	8/136 (5.9)	.782	1.17 (0.39-3.47)
Aortic reintervention	8/272 (2.9)	6/136 (4.4)	2/136 (1.5)	.179	0.33 (0.07-1.65)
Renal failure	29/272 (10.7)	17/136 (12.5)	12/136 (8.8)	.353	0.71 (0.34-1.48)
Respiratory failure	66/272 (24.3)	31/136 (22.8)	35/136 (25.7)	.579	1.17 (0.68-2.01)
True lumen expansion	91/255 (35.7)	37/129 (28.7)	54/126 (42.9)	.019	0.54 (0.31-0.93)
Neurologic complication	40/272 (14.7)	18/136 (13.2)	22/136 (16.2)	.481	1.29 (0.64-2.59)
Ischemic stroke	27/272 (9.9)	12/136 (8.8)	15/136 (11)		
Hemorrhagic stroke	5/272 (1.8)	4/136 (2.9)	1/136 (0.7)		
Paraplegia	3/272 (1.1)	0/136 (0)	3/136 (2.2)		
Others	5/272 (1.8)	2/136 (1.5)	3/136 (2.2)		

*HAR*, Hemiarch replacement; *TAR*, total arch replacement; *FET*, frozen elephant trunk.

**Figure 1:**
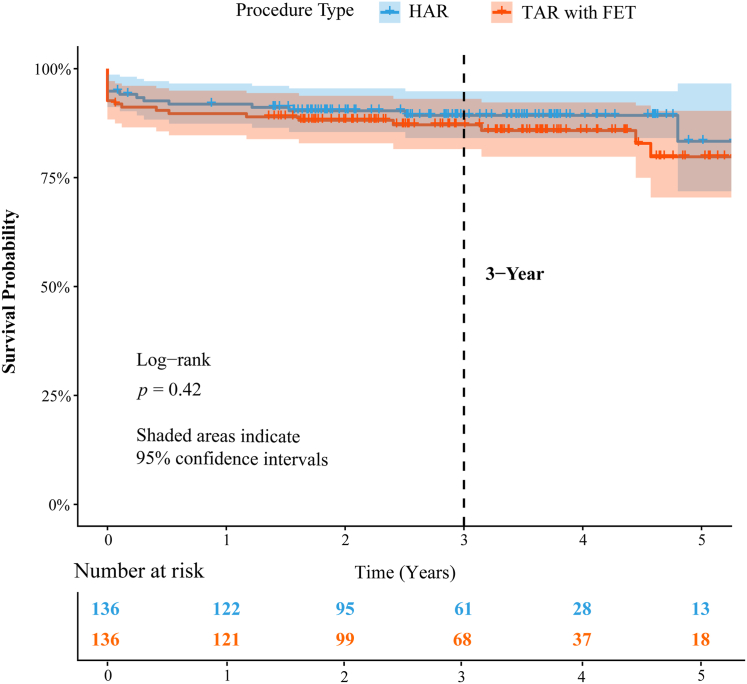
Kaplan-Meier survival curves comparing HAR and TAR with FET. *HAR*, Hemiarch replacement; *TAR*, total arch replacement; *FET*, frozen elephant trunk.

**Figure 2:**
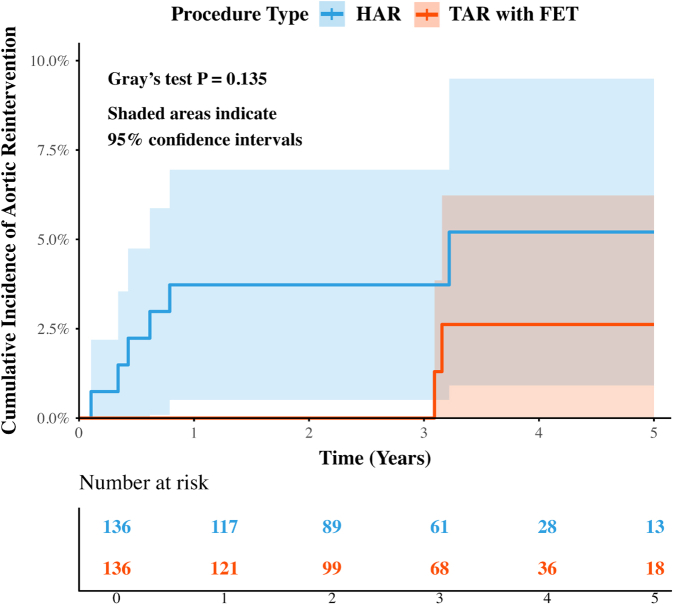
Cumulative incidence of aortic reintervention (competing risk analysis): HAR versus TAR with FET. *HAR*, Hemiarch replacement; *TAR*, total arch replacement; *FET*, frozen elephant trunk.

Kaplan-Meier analysis further showed no significant difference in the 3-year cumulative survival rate between the 2 groups (log-rank *P* = .42) (Figure 1). Considering death as a competing event, a competing risk model was used to evaluate the cumulative incidence of aortic reintervention, which also demonstrated no statistically significant difference between the groups (Gray test *P* = .135) (Figure 2).

Regarding secondary outcomes, the 2 groups had similar rates of re-exploration for bleeding (5.1% vs 5.9%, *P* = .782), renal failure (12.5% vs 8.8%; OR, 0.71; 95% CI, 0.34-1.48; *P* = .353), respiratory failure (22.8% vs 25.7%; OR, 1.17; 95% CI, 0.68-2.01; *P* = .579), and overall neurologic complications (13.2% vs 16.2%, *P* = .481).

In a subgroup analysis of patients presenting with acute malperfusion, postoperative mortality and major complications were increased overall (Table E4). However, no significant differences were observed between HAR and TAR with FET in operative mortality (7.1% vs 9.7%, *P* = .694) or major perioperative complications, including renal failure and neurologic complications.

**Figure 3:**
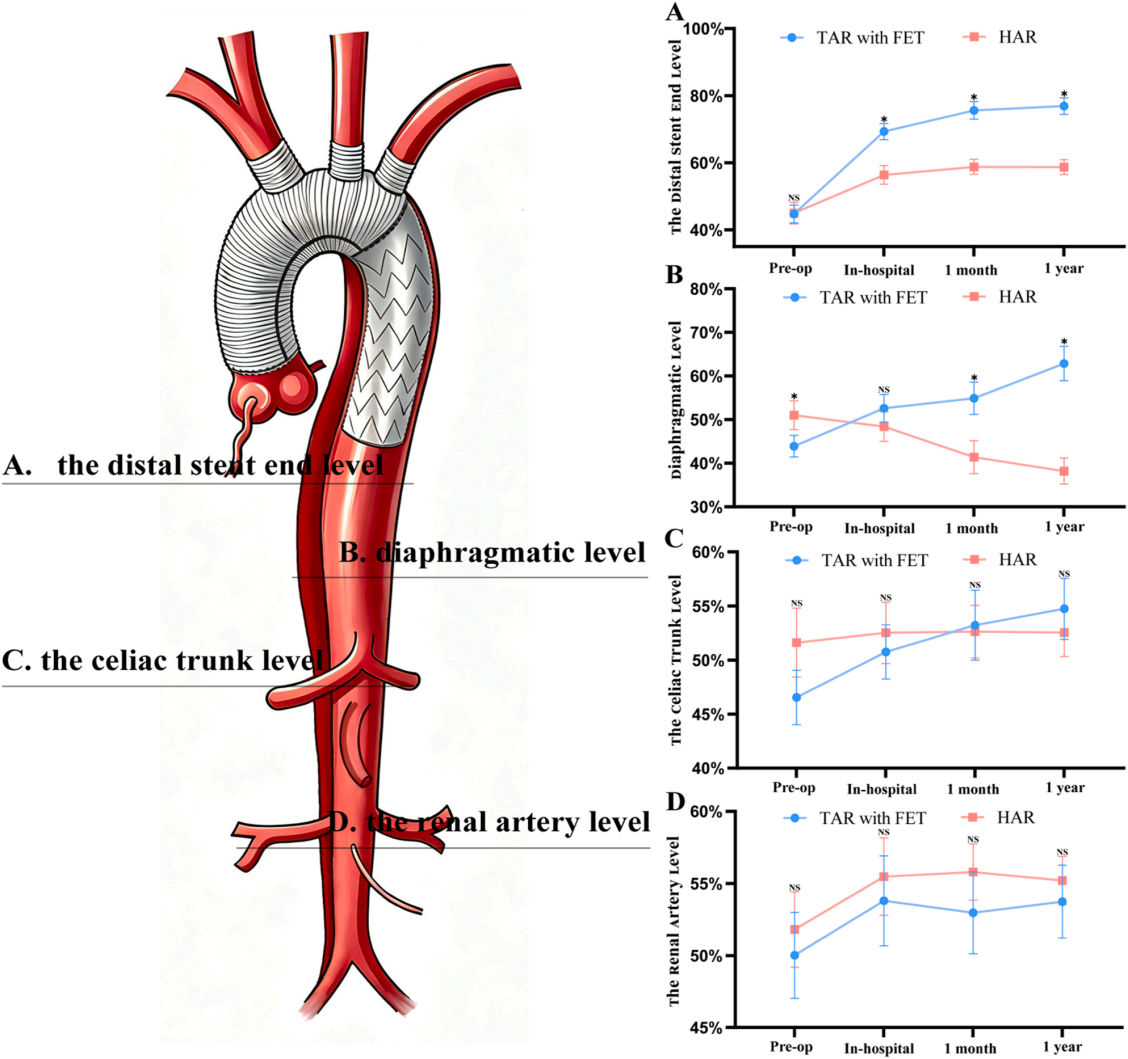
Superior aortic positive remodeling after TAR with FET at 4 anatomical levels: A, Stent end; B, Diaphragmatic; C, Celiac trunk; and D, Renal artery levels. The ratio was defined as the true lumen diameter divided by the sum of the true and false lumen diameters at each level. In the HAR group, the level corresponding to the distal stent end was determined at the anatomically matched descending aortic plane, located at the same distance from a predefined proximal landmark as the distal stent end in the TAR with FET group. *Error bars* indicate 95% CIs. ∗*P* < .05 was considered statistically significant between the 2 groups. *TAR*, Total arch replacement; *FET*, frozen elephant trunk; *HAR*, hemiarch replacement.

**Figure 4:**
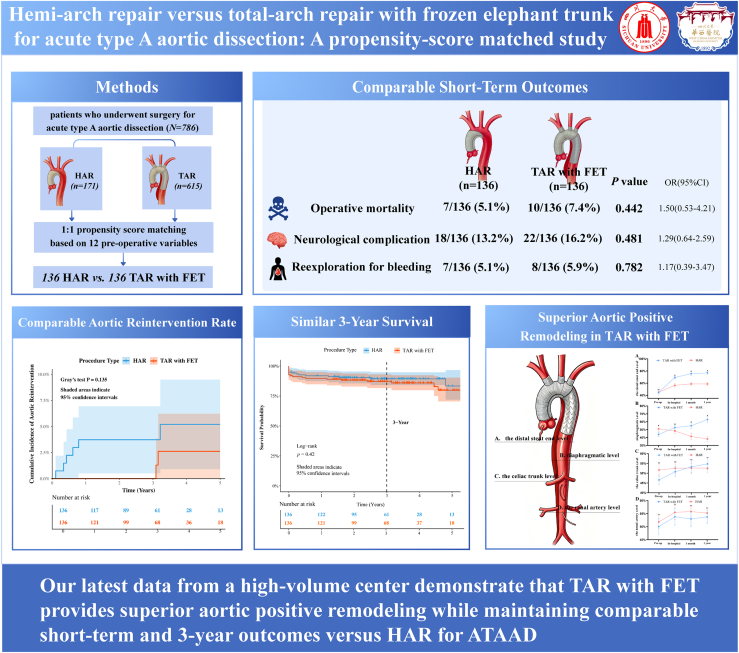
In propensity score-matched patients with acute type A aortic dissection, TAR with FET achieved similar perioperative outcomes, 3-year survival, and aortic reintervention rates but more favorable aortic remodeling than HAR. *TAR*, Total arch replacement; *FET*, frozen elephant trunk; *HAR*, hemiarch replacement.

Imaging evaluation revealed that the TAR with FET group exhibited significantly superior aortic positive remodeling compared with the HAR group. Specifically, the proportion of patients achieving true lumen expansion was significantly greater in the TAR with FET group (28.7% [37/129] vs 42.9% [54/126]; OR, 0.54; 95% CI, 0.31-0.93; *P* = .019). As further illustrated in Figure 3, the TAR with FET group demonstrated a greater true lumen/(true lumen + false lumen) ratio than the HAR group at both 1 month and 1 year postoperatively, indicating that TAR with FET is associated with better aortic remodeling effects in midterm follow-up. Detailed true lumen diameters are provided in Table E5. However, this difference was mainly observed at the distal stent end and diaphragmatic levels, whereas no significant between-group differences were identified at the more distal abdominal levels, including the celiac trunk and renal artery levels, during follow-up. The overall study design and key findings are summarized in Figure 4.

## Discussion

This propensity score–matched analysis compared the early and midterm outcomes of HAR versus TAR with FET in patients with ATAAD. After matching, multiple key surgical risk factors were well balanced between the 2 groups, such as age and previous cardiac surgery.^23^, ^24^, ^25^ No significant differences were observed between the 2 groups in terms of operative mortality, renal failure, respiratory failure, neurologic complications, or 3-year survival. Although TAR with FET showed a greater rate of true lumen expansion and better true lumen ratio at proximal descending aorta at 1-year follow-up, no significant difference in 3-year aortic reintervention rate was identified in our midterm follow-up.

This study provides the latest evidence from a high-volume aortic center in Asian on arch repair strategies for ATAAD. In the context of the present study, a “high-volume aortic center” refers to a specialized center with a dedicated aortic team, established perioperative management pathways, and sustained experience in managing a large number of ATAAD cases. In our center, in-hospital mortality was 5.1%, and neurologic complications occurred in 13.0%. Although mortality was relatively low, both rates were within the ranges reported by contemporary ATAAD series.^26^, ^27^, ^28^ In the matched cohort, ischemic stroke remained the predominant neurologic complication and occurred at a broadly similar frequency in the 2 groups (8.8% in the HAR group vs 11.0% in the TAR with FET group). Notably, all cases of paraplegia occurred in the TAR with FET group (0 vs 2.2%, 3 patients). Although the number of spinal cord injury events was too small to obtain any conclusion, this finding still highlighted the importance of continued refinement in spinal cord protection and distal aortic management, especially with application of FETs. Overall, our findings are consistent with recent reports from experienced high-volume aortic centers. In particular, Elbatarny and colleagues^18^ reported in a multicenter national registry that extended arch repair yielded perioperative outcomes comparable with those of hemiarch repair in acute type A aortic dissection, supporting the safety of a more extensive arch strategy in appropriately selected patients. In parallel, another recent study by Pendarvis and colleagues^29^ in April 2025 also supported that complex aortic arch reconstruction can provide a framework for subsequent distal aortic endovascular procedures and can be performed without increasing the risk of complications or mortality.

The evolution of this safety profile is further illustrated by longer-term data. A study spanning 1999-2019 demonstrated a clear change over time: Before 2009, TAR with FET was associated with a greater neurologic risk than HAR (OR, 3.28; 95% CI, 1.23-8.98; *P* = .017). However, after 2009, the neurologic risk between the 2 procedures became comparable (*P* = .13).^30^ Therefore, in experienced high-volume aortic centers, refinements in cerebral protection and surgical technique may help reduce the additional neurologic risk traditionally associated with TAR, thereby narrowing the gap with HAR.

Given this comparable safety, the balance of surgical decision-making tilts toward considerations of long-term efficacy. Our study, along with increasing high-level evidence, suggests that TAR with FET offers theoretical and anatomical advantages in promoting false lumen thrombosis and favorable aortic remodeling.^31^^,^^32^ The latest Society for Vascular Surgery/Society of Thoracic Surgeons aortic dissection classification study (2025) provides a theoretical foundation for this, indicating that dissections with the primary entry tear located in the aortic arch (Society for Vascular Surgery/Society of Thoracic Surgeons type B0) are associated with a significantly greater rate of distal aortic reintervention.^33^ This strengthens the rationale for opting for a more extensive TAR with FET during the index operation in such patients, to eliminate the arch entry tear and thereby potentially reduce the risk of future distal reinterventions.^16^

Compared with regions such as Europe and America, patients undergoing surgery for ATAAD in China are generally younger. Patients who are Chinese experience disease onset approximately 10 years earlier compared with data from the International Registry of Acute Aortic Dissection.^13^ For patients with ATAAD, the TAR combined with FET procedure is adopted more frequently in China. This may be attributed to its nearly 2-decade history of application in the country.^4^^,^^7^^,^^13^ Although contemporary arch management for ATAAD has become increasingly diverse, including HAR, zone 2 arch replacement with or without endovascular repair, and staged hybrid strategies, the choice of arch strategy should be based on the extent of the dissection and the distal aortic anatomy. Some recent reports have suggested that, in patients with more extensive aortic involvement, FET implantation during the operation may be more advantageous.^14^ By extending treatment into the proximal descending aorta, FET may help seal distal entry tears, promote false lumen thrombosis and true lumen expansion, and facilitate future endovascular intervention, if needed. These potential advantages have been reported not only with Sun's procedure but also with widely used FET devices such as Thoraflex Hybrid and E-vita.^14^^,^^34^^,^^35^ However, whether these different arch strategies lead to meaningful differences in long-term survival and aortic reintervention remains uncertain and requires further evaluation in larger studies with longer follow-up.

Despite the superior aortic remodeling observed in the TAR with FET group, the reintervention rates between the 2 groups were similar during midterm follow-up. This inconsistency can be explained by several factors. First, the current 3-year follow-up period might be insufficient to capture the long-term clinical consequences of differential aortic remodeling. Second, the reintervention rate for residual dissection in the descending aorta is relatively low. Even with negative remodeling, the pathology may not have progressed to meet intervention criteria, or patients might defer surgery.^36^ In addition, our multiplanar imaging analysis showed that the remodeling advantage of TAR with FET was not uniform along the entire aorta. The difference between the 2 groups was mainly observed at the more proximal levels, where TAR with FET was associated with a significantly greater true lumen/(true lumen + false lumen) ratio during follow-up. In contrast, no clear differences were identified at the more distal abdominal levels, including the celiac trunk and renal artery levels. These findings suggest that the beneficial remodeling effect of TAR with FET may be more pronounced in the proximal descending thoracic aorta, whereas its influence on the distal abdominal aorta is more limited. This regional pattern may partly explain why the superior proximal remodeling observed after TAR with FET did not translate into a significant difference in mid-term aortic reintervention between the 2 groups.

In this study, there were 6 patients who required reintervention in the HAR group and 2 in the TAR with FET group. Within the HAR group, reoperations mainly targeted progressive false lumen dilatation related to residual dissection or new entry tears. Specifically, 5 patients underwent thoracic endovascular aortic repair with endovascular reconstruction of the supra-aortic branches because of false lumen enlargement, whereas 1 patient underwent reoperative TAR with FET. In contrast, both reoperations in the TAR with FET group occurred in the abdominal aorta, distal to the initial surgical extent, presenting as dissecting aneurysms or persistent false lumen perfusion. These findings suggest different patterns of reintervention between the 2 groups, whereas continued follow-up of the distal aorta is required after either strategy. Moreover, connective tissue disorders such as Marfan syndrome may be associated with an increased risk of reoperation. In this cohort, patients with Marfan syndrome constituted 2.2% (6/272) of the total population but accounted for 25% (2/8) of the reoperation cases. Specifically, the reoperation rate among patients with Marfan syndrome was 50% (1/2) after HAR and 25% (1/4) after TAR with FET. Although the small number of patients with Marfan syndrome in this cohort limits statistical power and potential statistical bias may exist, this observation still suggests that more extensive arch replacement might yield superior long-term outcomes in patients with such high-risk factors.

On the basis of these findings, we believe that future selection of surgical strategies for the aortic arch should be guided by more rigorous indications. This includes developing more precise treatment strategies for specific patient subgroups, such as those with uncomplicated ATAAD.^37^ Therefore, our registered randomized controlled trial (ChiCTR2500114775) comparing HAR versus TAR with FET in uncomplicated acute DeBakey I aortic dissection is ongoing. In parallel, a Canadian multicenter randomized trial, TITAN:HEADSTART (NCT03885635), is also comparing hemiarch versus extended arch repair in patients with acute DeBakey type I aortic dissection, further underscoring that the optimal extent of repair in ATAAD remains unresolved and requires prospective randomized evidence.^38^

### Limitations

This study has several limitations. First, as a retrospective, single-center study, it carries an inherent risk of selection bias. The perioperative outcomes observed in our study may partly reflect the experience of a high-volume center and should be generalized with caution to centers with different operative volumes or less-established expertise. Second, although propensity-score matching was used to balance intergroup differences, some unmeasured confounding factors may still exist. Third, the number of key outcome events, particularly reinterventions, was limited, which reduced the statistical power to detect clinically meaningful differences between the two strategies. Therefore, the absence of a statistically significant difference in reintervention should not be interpreted as evidence of equivalence between the 2 approaches. Fourth, the follow-up period was relatively short (median, 3 years), which is insufficient to adequately assess late differences in survival or reintervention between the 2 strategies. Longer follow-up is required to confirm the potential advantage of TAR with FET in preventing long-term aortic events. Finally, in this study, aortic remodeling was assessed using the true-to-false lumen ratio measured at several specific anatomical levels on postoperative CTA. Although widely applied, this provides only a rough reflection of overall aortic changes. Future studies would benefit from richer imaging follow-up data (eg, 3-dimensional volumetric measurements, false lumen thrombosis grading) to refine the assessment of long-term outcomes.

## Conclusions

Among patients with ATAAD, both HAR and TAR with FET are associated with comparable perioperative outcomes and 3-year survival. Although TAR with FET demonstrates more favorable aortic remodeling, this anatomical advantage does not translate into a significant difference in the reintervention rate in our follow-up period. Our findings suggest that TAR with FET may be a reasonable surgical option in selected patients treated at high-volume aortic centers. Further investigation through prospective studies with prolonged follow-up is warranted to establish optimal indications of TAR with FET for ATAAD repair.

## Conflict of Interest Statement

The authors reported no conflicts of interest.

The *Journal* policy requires editors and reviewers to disclose conflicts of interest and to decline handling or reviewing manuscripts for which they may have a conflict of interest. The editors and reviewers of this article have no conflicts of interest.

## Figures and Tables

**Table E1:** Preoperative variables used for propensity score matching

Variable	Total (n = 272)	HAR (n = 136)	TAR with FET (n = 136)	*P* value	SMD
Age, y, mean (SD)	54.58 (11.06)	54.96 (11.85)	54.19 (10.23)	.415	0.070
Hypertension, n (%)	202 (74.3)	100 (73.5)	102 (75)	.890	0.034
Renal insufficiency, n (%)	17 (6.2)	8 (5.9)	9 (6.6)	1.000	0.030
Marfan syndrome, n (%)	6 (2.2)	2 (1.5)	4 (2.9)	.683	0.100
D-dimer, mg/L FEU, median [Q1, Q3]	5.74 [2.36, 13.62]	5.68 [2.04, 12.53]	5.91 [2.71, 14.23]	.675	0.008
BNP, ng/L, median [Q1, Q3]	468 [183.25, 1096]	448.5 [171.25, 1163.5]	477 [192.75, 1061.75]	.953	0.011
Preoperative hypotension, n (%)	26 (9.6)	14 (10.3)	12 (8.8)	.831	0.050
Preoperative altered mental status, n (%)	30 (11)	16 (11.8)	14 (10.3)	.850	0.047
Preoperative limb ischemia, n (%)	32 (11.8)	18 (13.2)	14 (10.3)	.571	0.091
Retrograde aortic dissection, n (%)	7 (2.6)	3 (2.2)	4 (2.9)	1.000	0.046
Pericardial tamponade, n (%)	16 (5.9)	8 (5.9)	8 (5.9)	1.000	0.000
Entry tear, n (%)					
Ascending aorta	226 (83.1)	113 (83.1)	113 (83.1)	1.000	0.000
Aortic arch	32 (11.8)	17 (12.5)	15 (11)	.823	0.046
Descending aorta	8 (2.9)	4 (2.9)	4 (2.9)	1.000	0.000
No identifiable entry tear	6 (2.2)	2 (1.5)	4 (2.9)	.617	0.100

*HAR*, Hemiarch replacement; *TAR*, total arch replacement; *FET*, frozen elephant trunk; *SMD*, standardized mean difference; *FEU*, fibrinogen equivalent units; *Q1*, first quartile; *Q3*, third quartile; *BNP*, B-type natriuretic peptide.

**Table E2:** Patient characteristics and perioperative outcomes in the unmatched cohort (before propensity score matching)

Variable	Total (n = 786)	Hemiarch (n = 171)	Total arch (n = 615)	*P* value
Sex, male, n (%)	601 (76.4)	106 (61.8)	495 (80.4)	<.001
Age, y, median [Q1, Q3]	51 [44, 59]	60 [50, 67.25]	50 [42, 57]	<.001
BMI, median [Q1, Q3]	25.18 [22.65, 27.775]	24.97 [22.3, 26.97]	25.26 [22.86, 28.08]	.129
Hypertension, n (%)	564 (71.8)	132 (77.0)	432 (70.2)	.084
Smoking, n (%)	306 (38.9)	48 (28.1)	258 (42.0)	<.001
Previous cardiac surgery, n (%)	119 (15.1)	34 (19.7)	85 (13.8)	.056
Renal insufficiency, n (%)	48 (6.1)	10 (5.9)	38 (6.1)	.929
Diabetes, n (%)	24 (3.1)	10 (5.9)	14 (2.3)	.017
Marfan syndrome, n (%)	20 (2.5)	2 (1.3)	18 (2.9)	.252
Coronary artery disease, n (%)	14 (1.8)	5 (2.6)	9 (1.5)	.298
Stroke, n (%)	25 (3.2)	5 (2.6)	20 (3.3)	.68
Valvular heart disease, n (%)	82 (10.4)	24 (13.8)	58 (9.4)	.096
COPD, n (%)	30 (3.8)	10 (5.9)	20 (3.2)	.104
Thyroid disease, n (%)	10 (1.3)	3 (2.0)	7 (1.1)	.355
Arrhythmia, n (%)	20 (2.5)	6 (3.3)	14 (2.3)	.483
Preoperative hypotension, n (%)	110 (14.0)	16 (9.2)	94 (15.3)	.043
Preoperative altered mental status, n (%)	81 (10.3)	23 (13.2)	58 (9.4)	.155
Preoperative limb ischemia, n (%)	145 (18.5)	25 (14.5)	120 (19.6)	.129
Retrograde aortic dissection, n (%)	23 (3.0)	1 (0.7)	22 (3.6)	.045
Pericardial tamponade, n (%)	40 (5.1)	10 (5.9)	30 (4.8)	.572
D-dimer, mg/L FEU, median [Q1, Q3]	6.89 [2.87, 14.985]	5.74 [2.3175, 13.7675]	7.19 [3.17, 16.16]	.113
Uric acid, median [Q1, Q3]	362 [280.5, 448]	340 [251, 428.25]	367 [290, 454]	.012
Creatinine, μmol/L, median [Q1, Q3]	91 [72, 117]	88 [67, 111.75]	91 [74, 117]	.109
BNP, ng/L, median [Q1, Q3]	418 [175, 968]	487 [188, 1431.75]	401 [165, 900]	.075
CRP, median [Q1, Q3]	56.5 [14.8, 106]	44.6 [14.175, 86.6]	58.4 [14.8, 108]	.294
Left ventricle, mm, median [Q1, Q3]	49 [46, 53]	48.5 [45, 52]	50 [46, 53]	.033
Operation duration, h, median [Q1, Q3]	7 [6.115, 8]	6.55 [5.5, 7.5]	7 [6.3, 8.05]	<.001
Aortic root reconstruction, n (%)	410 (52.2)	78 (45.4)	332 (54.0)	.045
Aortic root replacement, n (%)	285 (36.2)	71 (41.4)	214 (34.8)	.111
Concomitant CABG, n (%)	73 (9.3)	16 (9.2)	57 (9.3)	.96
Core temperature, °C, median [Q1, Q3]	24 [23.3, 24.7]	23.8 [22.975, 25]	24 [23.4, 24.5]	.426
Cardiopulmonary bypass time, min, median [Q1, Q3]	193 [165, 235.5]	181 [150, 226.25]	198 [172, 239]	<.001
Crossclamp time, min, median [Q1, Q3]	142 [113.5, 177.5]	117 [100, 168]	146 [120, 184]	<.001
Fresh frozen plasma transfused, units, median [Q1, Q3]	0 [0, 200]	0 [0, 0]	0 [0, 300]	.201
Maximum intraoperative blood glucose, mmol/L, median [Q1, Q3]	10.3 [8.6, 12.4]	10.15 [8.2, 12.525]	10.4 [8.8, 12.4]	.553
Maximum intraoperative lactate level, mmol/L, median [Q1, Q3]	5.5 [3.9, 8.5]	5.7 [3.5, 8.15]	5.5 [4, 8.4]	.338
Circulatory arrest time, min, median [Q1, Q3]	25 [19, 31]	22 [16, 28.25]	27 [22, 33]	<.001
Operative mortality, n (%)	40 (5.1)	12 (7.2)	28 (4.5)	.148
Neurologic complication, n (%)	102 (13.0)	29 (17.1)	73 (11.8)	.071

*BMI*, Body mass index; *Q1*, first quartile; *Q3*, third quartile; *COPD*, chronic obstructive pulmonary disease; *FEU*, fibrinogen equivalent units; *BNP*, B-type natriuretic peptide; *CRP*, C-reactive protein; *CABG*, coronary artery bypass grafting.

**Table E3:** Frozen elephant trunk graft characteristics

Diameter × length (mm × mm)	Patients, n (%)
26 × 120	17/136 (12.50)
24 × 120	48/136 (35.29)
26 × 100	38/136 (27.94)
24 × 100	33/136 (24.26)

**Table E4:** Subanalysis of patients presenting with malperfusion

Variable, n (%)	Total (n = 73)	Hemiarch (n = 42)	Total arch (n = 31)	*P* value
Operative mortality	6/73 (8.2)	3/42 (7.1)	3/31 (9.7)	.694
Re-exploration for bleeding	3/73 (4.1)	1/42 (2.4)	2/31 (6.5)	.571
Renal failure	18/73 (24.7)	11/42 (26.2)	7/31 (22.6)	.789
Respiratory failure	29/73 (39.7)	14/42 (33.3)	15/31 (48.4)	.231
Neurologic complication	15/73 (20.5)	7/42 (16.7)	8/31 (25.8)	.389

**Table E5:** True lumen diameter at different aortic levels

Time point	The distal stent end level			Diaphragmatic level			The celiac trunk level			The renal artery level		
	Hemiarch	Total arch	*P* value	Hemiarch	Total arch	*P* value	Hemiarch	Total arch	*P* value	Hemiarch	Total arch	*P* value
Preoperative	1.25 (0.60)	1.32 (0.52)	.320	1.41 (0.59)	1.27 (0.42)	.030	1.34 (0.53)	1.23 (0.42)	.067	1.15 (0.38)	1.11 (0.46)	.450
In-hospital	1.57 (0.63)	1.85 (0.44)	<.001	1.46 (0.57)	1.59 (0.53)	.060	1.37 (0.49)	1.34 (0.48)	.622	1.24 (0.41)	1.20 (0.48)	.475
1-mo postoperative	1.69 (0.50)	1.90 (0.44)	<.001	1.51 (0.61)	1.71 (0.62)	.010	1.45 (0.47)	1.41 (0.51)	.516	1.23 (0.26)	1.19 (0.41)	.355
1-y postoperative	1.83 (0.42)	1.95 (0.44)	.027	1.54 (0.67)	1.84 (0.72)	<.001	1.48 (0.41)	1.48 (0.46)	1.000	1.33 (0.56)	1.23 (0.54)	.148
